# The use of high-throughput sequencing to investigate an outbreak of glycopeptide-resistant Enterococcus faecium with a novel quinupristin-dalfopristin resistance mechanism

**DOI:** 10.1007/s10096-018-3214-x

**Published:** 2018-02-24

**Authors:** Timothy D. Shaw, D. J. Fairley, T. Schneiders, M. Pathiraja, R. L. R. Hill, G. Werner, J. S. Elborn, R. McMullan

**Affiliations:** 1 Centre for Experimental Medicine, Queen’s University Belfast, Belfast, Northern Ireland UK; 2 Department of Medical Microbiology, Kelvin Laboratory, Royal Victoria Hospital, Belfast, Northern Ireland UK; 3 Division of Infection and Pathway Medicine, University of Edinburgh Medical School, Edinburgh, Scotland UK; 4 Antimicrobial Resistance & Healthcare-Associated Infection, Public Health England, London, UK; 5 National Reference Centre for Staphylococci and Enterococci, Robert Koch-Institute, Wernigerode Branch, Berlin, Germany; 6 National Heart and Lung Institute, Imperial College London, London, UK

**Keywords:** Quinupristin-dalfopristin, Enterococcus faecium, Enterococcal resistance, High-throughput sequencing, Outbreak

## Abstract

High-throughput sequencing (HTS) has successfully identified novel resistance genes in enterococci and determined clonal relatedness in outbreak analysis. We report the use of HTS to investigate two concurrent outbreaks of glycopeptide-resistant *Enterococcus faecium* (GRE) with an uncharacterised resistance mechanism to quinupristin-dalfopristin (QD). Seven QD-resistant and five QD-susceptible GRE isolates from a two-centre outbreak were studied. HTS was performed to identify genes or predicted proteins that were associated with the QD-resistant phenotype. MLST and SNP typing on HTS data was used to determine clonal relatedness. Comparative genomic analysis confirmed this GRE outbreak involved two distinct clones (ST80 and ST192). HTS confirmed the absence of known QD resistance genes, suggesting a novel mechanism was conferring resistance. Genomic analysis identified two significant genetic determinants with explanatory power for the high level of QD resistance in the ST80 QD-resistant clone: an additional 56aa leader sequence at the N-terminus of the *lsaE* gene and a transposon containing seven genes encoding proteins with possible drug or drug-target modification activities. However, HTS was unable to conclusively determine the QD resistance mechanism and did not reveal any genetic basis for QD resistance in the ST192 clone. This study highlights the usefulness of HTS in deciphering the degree of relatedness in two concurrent GRE outbreaks. Although HTS was able to reveal some genetic candidates for uncharacterised QD resistance, this study demonstrates the limitations of HTS as a tool for identifying putative determinants of resistance to QD.

## Introduction

Enterococci are Gram-positive, facultatively anaerobic cocci that naturally colonise the human gastrointestinal tract and commonly cause nosocomial infections [1]. In February 2017, the World Health Organization named *E. faecium* in a priority list of pathogens to guide their strategy for tackling antimicrobial resistance [2].

Quinupristin-dalfopristin (QD) is a semi-synthetic 70:30 mixture of streptogramins A and B licenced for treatment of glycopeptide-resistant *Enterococcus faecium* (GRE) and methicillin-resistant *Staphylococcus aureus* (MRSA) infection [3]. Quinupristin and dalfopristin act synergistically to inhibit the ribosomal peptide elongation cycle with bactericidal effect.

QD resistance in *E. faecium* is uncommon but several of its genetic determinants have been well-described [4, 5]. In most cases, high-level QD resistance (MIC ≥ 4 μg/ml) is conferred by enzymatic drug acetylation (encoded by the *vat* genes, particularly *vatD* and *vatE*) or drug efflux (encoded by *vgaA*, *vgaB* and *vgaD*) of quinupristin. The *vat* and *vga* families of genes are characteristically plasmid-borne and have been described in other Gram-positive organisms, mainly staphylococci [5]. Targeting of dalfopristin by resistance gene products may also be associated with low-level QD resistance (MIC < 4 μg/ml), with described mechanisms including drug degradation (*vgbA* and *vgbB*), efflux (*msrC*) and methylation of the 23S rRNA target site (*ermB*).

The discovery of novel genes and allele variants within the already-described families of QD resistance genes has led to speculation that there remain undiscovered genetic determinants of resistance [6, 7]. High-throughput sequencing (HTS) of enterococci has successfully identified undescribed genes of resistance, including those targeting linezolid, daptomycin and tigecycline [8–10]. HTS has also been useful in elucidating complex transmission routes in nosocomial outbreaks of GRE [11].

Two concurrent single-ward outbreaks of GRE were reported in Belfast in 2012. The two wards (ward A, a Haematology Unit, and ward B, a Critical Care Unit) were situated around 1 km apart within the same Hospital Trust. There is frequent movement of healthcare workers and patients between the two sites and between the two units. In response to the initial cases, GRE screening of new admissions to ward A and ward B was instigated until resolution of the outbreak.

GRE isolates from the outbreak were characterised by displaying low- or high-level resistance to QD. Genetic testing at the Public Health England Antimicrobial Resistance & Healthcare-Associated Infection (ARHAI) Reference Unit indicated the absence of *vatD* and *vatE* genes in the high-level QD-resistant GRE isolates. The impression from epidemiological and antibiogram analysis was that these isolates represented a Trust-wide outbreak. We hypothesised that a novel genetic mechanism was conferring high-level QD resistance in these isolates of GRE and sought to detect this mechanism using HTS. We also postulated that HTS could determine whether the two outbreaks were epidemiologically linked.

## Materials and methods

### Isolation

Enterococcal isolates were cultured from faeces of patients from two wards (ward A and ward B) in Belfast hospitals in the course of screening for carriage of GRE in an outbreak setting. The minimum inhibitory concentration (MIC, μg/ml) of QD was initially determined using an automated method (VITEK-2®, bioMérieux) in the local laboratory and confirmed using agar dilution (BSAC method) at the Public Health England reference laboratory. The QD MIC breakpoint for resistance was defined as ≥ 4 μg/mL. Seven QD-resistant isolates of *E. faecium* that were representative of both wards over a 6-month period were selected for study. Five QD-susceptible isolates of GRE, including one sporadic GRE isolate from a Hospital Trust outside Belfast (ward C), cultured during the same screening exercise, were used as control strains.

### QD-susceptibility testing

All *E. faecium* isolates that were QD-resistant or QD-susceptible by the agar dilution (BSAC method) at Public Health England were re-tested for QD-susceptibility using VITEK-2® (bioMérieux) in the local laboratory to confirm the persistence of phenotypic resistance prior to DNA extraction for HTS.

### PCR detection of genes of interest

The presence of genes conferring resistance to QD (*vatA, vatB, vatC, vatD, vatE, vatH, vgaA, vgaB, vgaD, vgbA, vgbB, msrC* and *ermB*) in the 12 isolates was assessed by PCR amplification using primers and PCR conditions as previously published (Table 1) [6, 12, 13]. Genomic DNA was extracted using the PureLink® Genomic DNA Purification Kit (Invitrogen). The positive control organisms for PCR were *S. aureus* ES1767, *S. aureus* ES1768, *E. faecium* UW1965 and *E. faecium* UW3540. Sterile water was used as a negative control.

**Table 1:** Primers and conditions for the detection of known QD resistance genes by PCR

Gene	Primers	Sequence	GenBank accession no.	Size of product (bp)	PCR conditions
VatA	Vat-1	TGGAGTGTGACAAGATAGGC	L07778	512	Initial denaturing reaction at 94 °C for 3 min; 35 cycles at 94 °C for 1 min, 55 °C for 1 min and 72 °C for 1 min; final extension reaction at 72 °C for 10 min
	Vat-2	GTGACAACAGCTTCTGCAGC			
VatB	vatB-1	GGCCCTGATCCAAATAGCAT	U19459	558	Initial denaturing reaction at 94 °C for 3 min; 35 cycles at 94 °C for 1 min, 55 °C for 1 min and 72 °C for 1 min; final extension reaction at 72 °C for 10 min
	vatB-2	GTGCTGACCAATCCCACCAT			
VatC	vatC-O	ATGAATTCGCAAAATCAGCAAGG	AF015628	579	Initial denaturing reaction at 95 °C for 3 min and 60 °C for 2 min; 30 cycles at 72 °C for 20 s, 95 °C for 20 s and 60 °C for 20 s; final extension reaction at 72 °C for 1 min
	vatC-P	TCGTCTCGAGCTCTAGGTCC			
VatD	satA-1	GCTCAATAGGACCAGGTGTA	L12033	271	Initial denaturing reaction at 94 °C for 3 min; 35 cycles at 94 °C for 1 min, 55 °C for 1 min and 72 °C for 1 min; final extension reaction at 72 °C for 10 min
	satA-2	TCCAGCTAACATGTATGGCG			
VatE	satG-1	ACTATACCTGACGCAAATGC	AF139725	511	Initial denaturing reaction at 94 °C for 3 min; 30 cycles at 94 °C for 20 s, 52 °C for 40 s and 72 °C for 50 s; final extension reaction at 72 °C for 6 min
	satG-2	GGTTCAAATCTTGGTCCG			
vgaA	vga-1	AGTGGTGGTGAAGTAACACG	M90056	659	Initial denaturing reaction at 94 °C for 3 min; 35 cycles at 94 °C for 1 min, 55 °C for 1 min and 72 °C for 1 min; final extension reaction at 72 °C for 10 min
	vga-2	CTTGTCTCCTCCGCGAATAC			
vgaB	vgaB-1	TGACAATATGAGTGGTGGTG	U82085	576	Initial denaturing reaction at 94 °C for 3 min; 35 cycles at 94 °C for 1 min, 52 °C for 1 min and 72 °C for 1 min; final extension reaction at 72 °C for 10 min
	vgaB-2	GCGACCATGAAATTGCTCTC			
vgbA	vgb-1	TACAGAGTACCCACTACCGA	M36022	569	Initial denaturing reaction at 94 °C for 3 min; 35 cycles at 94 °C for 1 min, 52 °C for 1 min and 72 °C for 1 min; final extension reaction at 72 °C for 10 min
	vgb-2	TCAATTCCTGCTCCAGCAGT			
vgbB	vgbB-Q	CAGCAGTCTAGATCAGAGTGG	AF015628	728	Initial denaturing reaction at 95 °C for 3 min and 60 °C for 2 min; 30 cycles at 72 °C for 20 s, 95 °C for 20 s and 60 °C for 20 s; final extension reaction at 72 °C for 1 min
	vgbB-R	CATACGGATCCATCTTTTCC			
ermB1	ermB-1	CATTTAACGACGAAACTGGC	M11180	424	Initial denaturing reaction at 94 °C for 3 min; 25 cycles at 94 °C for 1 min, 52 °C for 1 min and 72 °C for 1 min; final extension reaction at 72 °C for 10 min
	ermB-2	GGAACATCTGTGGTATGGCG			
vatH	vatG1	GTGGGAAAAGCATACACCT	GQ205627	200	Initial denaturing reaction at 94 °C for 5 min; 30 cycles at 94 °C for 30 s, 55 °C for 30 s, and 72 °C for 30 s; final extension reaction at 72 °C for 10 min
	vatG2	TTGCAGGATTACCACCAAC			
vgaD	vgaD1	CAACTGGAGCGAGCTGTTA	GQ205627	201	Initial denaturing reaction at 94 °C for 5 min; 30 cycles at 94 °C for 30 s, 55 °C for 30 s, and 72 °C for 30 s; final extension reaction at 72 °C for 10 min
	vgaD2	GACAGCCGGATAATCTTTTG			
msrC	msrC3	AAGGAATCCTTCTCTCTCCG	AJ243209	343	Initial denaturing reaction at 95 °C for 3 min; 35 cycles 93 °C for 30 s, and 55 °C for 2 min at 72 °C for 90 s; final extension reaction at 72 °C for 10 min
	msrC4	GTAAACAAAATCGTTCCCG			

### High-throughput sequencing

Sequencing libraries were prepared using the Nextera® DNA Library Prep Kit (Illumina) according to the manufacturer’s instructions. The target insert size for libraries was 500 bp. Libraries were sequenced using the HiSeq 2000 sequencing system (Illumina, San Diego, CA) using 90 bp paired-end sequencing mode at the Beijing Genome Institute. Sequence data quality was assessed using FastQC software (http://www.bioinformatics.babraham.ac.uk/projects/fastqc). All sequences were reported to be high quality, with median Phred quality scores above 30 across all bases for all samples.

### De novo assembly

For each sample, paired-end de novo assembly was undertaken using clc_assembler software (http://www.clcbio.com/products/clc-assembly-cell) with differing parameters. Limiting the output to contigs of 200 or more bases, 7 different insert size ranges were specified: 120–500; 170–500; 220–500; 270–500; 320–500; 370–500 and 420–500. The optimal assembly for each sample was defined as the assembly containing the largest contig. These optimal assemblies were selected for further analysis.

The *Enterococcus faecium* DO genome was used as a reference for contig ordering and for comparison to the study strains. This strain is susceptible to QD (MIC ≤ 1 μg/mL). The DO genome comprises a chromosome (NC_017960, 2,698,137 bp) and three plasmids (NC_017961, plasmid 1, 36,262 bp; NC_017962, plasmid 2, 66,247 bp and NC_017963, plasmid 3, 251,926 bp). For each sample, contigs were ordered using BLASTN comparison to the DO genome. Contig order relative to the DO genome was used to generate a reordered assembly for each sample. Contigs that did not align to the DO reference were placed first in the reordered assembly. Gaps between contigs which were ‘size-unknown’ were indicated with a block of 100 N residues. Other N residues in the assemblies indicated of gaps of known length, based on read-pair insert size distributions.

### Annotation

Identification of predicted CDS (coding segment) regions in the assemblies used a combination of two approaches: ab initio ORF finding using Artemis (http://www.sanger.ac.uk/science/tools/artemis) software and transitive annotation using BLAST from known *E. faecium* predicted proteins. A dataset (‘EfecDB’) comprising all 670,389 *E. faecium* predicted proteins was downloaded from NCBI. For each predicted CDS in each assembly, the EfecDB database was searched using BLASTP. The ‘most similar protein’ description line was used to assign a protein product to each predicted CDS.

### MLST alleles

MLST allele sequences (*atpA*, *ddl*, *gdh*, *purK*, *gyd*, *pstS* and *adk*) for *E. faecium* were downloaded from the PubMLST database (https://pubmlst.org/efaecium). These alleles were identified in assembled HTS data from the sequenced strains using blastn and the PubMLST batch sequence query tool. The resulting allelic profiles were used to assign sequence types.

### Genome-wide single nucleotide polymorphism analysis

HTS data were used to generate phylogenies based on single nucleotide polymorphism (SNP) analysis, using two different methods. The first method (described by Kaas et al.) was implemented on the Centre for Genomic Epidemiology server (https://cge.cbs.dtu.dk/services/CSIPhylogeny) [14]. The second method (described by Bertels et al.) was implemented on the REALPHY server v1.12 (https://realphy.unibas.ch/fcgi/realphy) [15].

### Specific genes of interest

To identify specific genes of interest in the assemblies, protein sequences were obtained from NCBI (see Appendix 1).

Each sequence was used to search all of the predicted protein sequences in the assemblies using BLASTP, allowing up to 500 hits. The outputs were parsed on the basis of hsp bitscore, a measure of similarity and extent of the similarity region, using a very stringent bitscore threshold of > 250. All hits were aligned with the query sequence and the resulting multiple sequence alignments were manually inspected using GeneDoc (http://www.nrbsc.org/old/gfx/genedoc).

### Comparative genomic analysis

Two rounds of analysis were undertaken to identify genes/predicted proteins that were associated with the QD-resistant phenotype. The first round identified predicted proteins in the assemblies that were present or absent from the DO reference genome, using BLASTP with a non-stringent 20% amino acid identity threshold. However, this analysis did not identify predicted proteins that were absent from the DO genome and only present in resistant strains. To address this, a second round of analysis was carried out, with reference to a ‘pan genome’ constructed using CDS data from all of the sequenced strains. This analysis aimed to identify homologous genes (proteins) that were present only in the assemblies from resistant strains and/or only present in resistant strains with the same MLST sequence type. A BLASTN threshold of 70% nucleotide identity was applied for this analysis.

## Results

### PCR detection of genes of interest

No PCR products were obtained using published primer sets for *vatA, vatB, vatC, vatD, vatE, vatH, vgaA, vgaB, vgaD, vgbA* and *vgbB* on genomic DNA extracted from any of the QD-resistant or QD-susceptible isolates. All of the tested isolates (both QD-resistant and sensitive) were PCR positive using *msrC* and *ermB* primers. For the positive controls, PCR products were seen at the expected band sizes for *S. aureus* ES1767 (v*atA, vgaA, vgbA*), *S. aureus* ES1768 (*vatB, vgaB*), *E. faecium* UW1965 (*vatD*) and *E. faecium* UW3540 (*vatE*).

### Multi-locus sequence typing (MLST) by HTS

MLST allele sequences (*atpA*, *ddl*, *gdh*, *purK*, *gyd*, *pstS* and *adk*) were identified in assembled HTS datasets for each strain and used to assign MLST sequence types. All seven alleles were identified in all of the sequenced strains, and all strains had known allelic profiles/sequence types. MLST demonstrated that the two concurrent outbreaks were each dominated by a single clone of QD-resistant *E. faecium:* four QD-resistant isolates were type ST80, of which three were isolated from ward A; three were ST192 and all isolated from ward B. One QD-susceptible isolate of *E. faecium* was also type ST192, with the other three QD-susceptible isolates having different ST types (Table 2).

**Table 2:** Genome assembly summary data for *E. faecium* isolates selected for high-throughput sequencing

Date isolated	Ward	Isolate	QD MIC at baseline	QD phenotype	MLST ST	Read depth	Contigs	Largest contig (bp)	Coverage (bp)	Coding sequences	Not present in DO genome
13/02/2012	A	Ent12	8	R	80	1,961,112	167	113,803	2,978,459	2976	128
20/03/2012	B	Ent8	8	R	192	1,950,000	183	120,337	3,051,329	3071	150
20/06/2012	A	Ent16	8	R	80	1,955,556	171	117,861	2,966,094	2965	125
25/06/2012	A	Ent9	8	R	80	1,961,112	165	109,771	2,955,813	2949	122
11/07/2012	B	Ent13	8	R	192	1,955,556	177	116,441	3,046,475	3048	152
19/07/2012	B	Ent36	8	R	192	1,950,000	176	120,328	2,997,011	2995	134
28/07/2012	B	Ent19	8	R	80	1,955,556	162	133,779	2,973,776	2941	137
02/04/2012	B	Ent22	1	S	192	1,961,112	166	132,639	2,994,545	2984	127
06/04/2012	B	Ent23	1	S	203	1,950,000	201	145,636	3,184,739	3241	170
07/05/2012	A	Ent28	1	S	262	1,961,112	371	189,792	3,192,843	3500	230
01/06/2012	C	Ent31	0.5	S	17	1,944,445	234	117,101	3,056,181	3099	173
20/06/2012	A	Ent34	1	S	78	1,955,556	163	224,457	3,033,536	3027	198

*MIC* minimum inhibitory concentration (μg/ml), *MLST* multi-locus sequencing type, *QDR* quinupristin-dalfopristin resistant, *QDS* quinupristin-dalfopristin susceptible, *S* susceptible, *I* intermediate resistance, *R* resistant; ward names have been anonymised

**Fig. 1:**
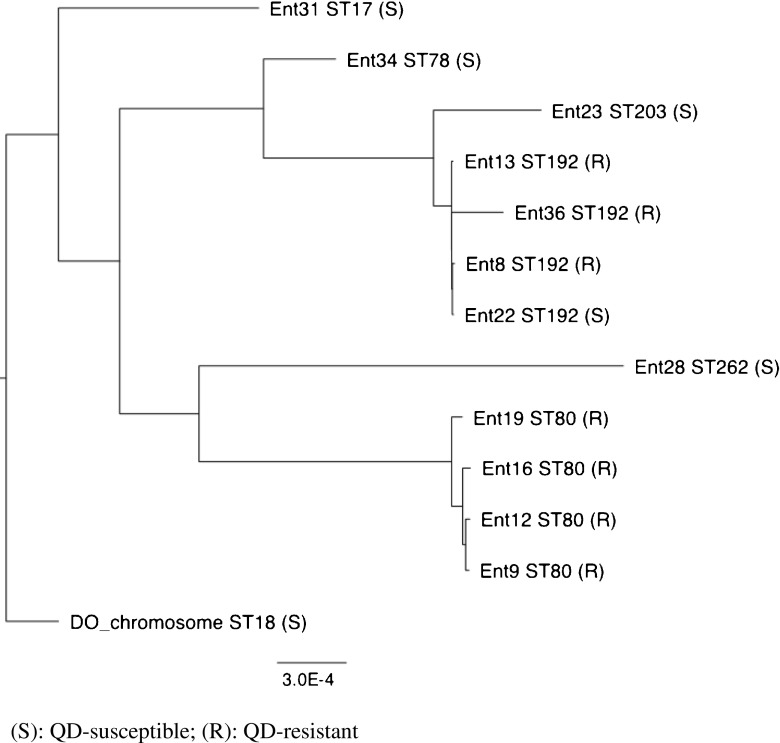
Cladogram depicting the clonal relatedness of Enterococcal isolates studied in this outbreak. S QD-susceptible, R QD-resistant

Genome-wide SNP analysis using two different software pipelines confirmed the strains that were designated as ST80 using MLST formed a single monophyletic cluster and were effectively indistinguishable from each other based on SNPs (Fig. 1).

### Comparative genomic analysis

Comparative genomic analysis was performed, comparing the assembled HTS datasets from the QD-resistant isolates (ST80 and ST192 strains), QD-susceptible isolates (all ST types) and the QD-sensitive *E. faecium* DO reference strain. The first comparison was to a list of genes encoding proteins of interest that are known to be associated with QD resistance. All of the sequenced strains contained *ermB*, *msrC* (as expected from the PCR data) and also *lsaE* homologues. However there were no genes (using at least 20% amino acid sequence similarity as a cutoff) belonging to the *vat* (*vatA, vatB, vatC, vatD, vatE, vatG*) or *vga* (*vgaA, vgaB, vgaD*) families identified in the QD-resistant or QD-susceptible isolates.

The second comparison was of all predicted protein-coding genes identified in the sequenced strains (*n* = 36,664 in total) to both the DO reference genome (*n* = 3114 genes) and to a ‘pan genome’ of sequences present in at least one of the sequenced strains, but not in the DO reference genome (*n* = 4131 genes). This comparison, using a less stringent cutoff (70% similarity at the DNA level) identified two genetic foci of interest.

First, a total of 68 predicted proteins were identified that were present only in the resistant ST80 strains. Fifty-five of the genes encoding these were located in eight syntenic clusters, corresponding to operons encoding saccharide metabolism, polysaccharide/capsule biosynthesis, or to transposons (identified by the presence of one or more genes annotated as a transposase). None of the 68 predicted proteins were annotated with functions that might confer QD resistance. The comparative analysis did identify a putative multidrug resistance transposon comprised of eight genes located in a single probable transposon in all of the ST80 strains. However, these genes were also present, with six additional flanking genes (HMPREF0351_12793, HMPREF0351_12806) as a transposon in the (QD susceptible) reference genome. Of note, two upstream genes to the transposon in the DO strain encode transcriptional regulators which were absent from the ST80 isolates: HMPREF0351_12795, a hypothetical WYL family DNA-binding transcriptional regulator (118 aa) and HMPREF0351_12796, a DNA-binding transcriptional regulator YafY containing HTH and WYL domains (190 aa). A further gene encoding a putative transcriptional repressor protein (HMPREF0351_12805) was present in the DO transposon, but absent from the ST80 assemblies. Analysis of this transposon, which may be de-repressed in the resistant ST80 isolate through the absence of the transcriptional regulators, predicted that it encodes several proteins with possible drug or drug-target modification activities. These include putative kanamycin kinase, streptothricin acetyltransferase, streptomycin aminoglycoside 6-adenyltransferase, SAM-dependent methyltransferase and rRNA (adenine-N(6)-)-methyltransferase enzymes. In addition, the transposon includes an unidentified DNA polymerase domain-containing protein and a small (81aa), unnamed hypothetical protein of unknown function belonging to the DUF1413 superfamily (http://pfam.xfam.org/family/PF07205).

The second observation of interest was an additional 56aa leader sequence at the N-terminus of the *lsaE* gene in the ST80 QD-resistant isolates that was not seen in the non-ST80 QD-resistant isolates, the QD-susceptible isolates or the DO reference strain. This gene encodes a homologue for the ATP-binding cassette (ABC) protein that is responsible for QD resistance in *E. faecalis* [16]. A point mutation in this gene (C1349T) has reportedly conferred QD resistance (MIC 4) to *E. faecium* [17]. However, this 56aa leader sequence has been found in another reference strain, *E faecium* TX1330, which we confirmed is QD-susceptible.

There were no unique genetic determinants found in the ST192 QD-resistant isolates that were absent from the ST80 QD-resistant isolates, the QD-susceptible isolates and/or the DO reference strain.

## Discussion

The potential of high-throughput sequencing is an exciting development in diagnostic bacteriology [18]. To date, the leading application of HTS has been determining clonal relatedness of bacterial isolates in outbreak analysis. However, as antimicrobial resistance is a growing priority in clinical research, HTS has been used to identify novel genes of interest. In enterococcal studies, HTS has already characterised previously unknown genes that confer resistance to linezolid, daptomycin and tigecycline [8–10]. This study sought to identify the novel genetic determinant for QD resistance in our isolates of GRE using HTS and determine the relatedness of two concurrent outbreaks. There are three principal findings from this study, with attendant implications.

First, PCR and HTS analysis confirmed that the QD-resistant *E. faecium* in this outbreak did not encode any known genes that confer high-level resistance to QD. This concurs with the recognition elsewhere that there remain uncharacterised QD resistance mechanisms among enterococci [6, 7]. Gene targets of interest that were not found using the search strategy employed may have been genuinely absent from the genomes, present in the genome but missing from the assemblies, or not detected due to divergence at the primary sequence level. We consider the latter to be unlikely as two Lsa-family ABC transporter variants that were distinct from—but clearly homologous to—the LsaE query sequence (amino acid sequences were 43% identical and 94% conserved physicochemical properties) were identified using this approach.

On account of the limitations of HTS and assembly strategy, none of the study strains was sequenced to closed genomes so we cannot exclude missing contigs which may harbour resistance determinants.

Currently, QD is not commonly used to treat serious GRE infections, as it has been superseded by agents that are easier to administer and are better tolerated. However, as GRE infections increase in number and acquire further drug resistance, we might expect to see greater dependence on QD in future clinical practice. Rapid resistance screening tests based on currently characterised resistance genes would have failed to detect QD resistance in some of these isolates implicated in this study. This has potential to delay the commencement of appropriate treatment, which could have adverse consequences for patient outcomes in the context of a QD-resistant GRE outbreak.

Second, HTS was useful in demonstrating the multi-clonal nature of this GRE outbreak. The benefit of typing outbreak strains for identifying infection source and transmission routes is well recognised [11, 18]. MLST typing of the HTS data revealed that two clones dominated this outbreak which linked strongly to the ward from which they were isolated: three of four ST80 isolates came from ward A and all four ST192 isolates came from ward B. This alerts infection control practitioners against presuming the association of outbreaks that appear linked in space and time, even if they share atypical features such as unusual antibiograms.

A number of ST80 strains (*n* = 17) from hospitalised patients and/or outbreaks in the UK, Europe, Asia and Africa are listed in the PubMLST *E. faecium* isolates database. The earliest ST80 strain in the database was isolated in Israel in 1997. ST80 is a single-locus variant of the globally disseminated multidrug-resistant ST117 clone and represents a further international hospital-adapted GRE clone [19]. To our knowledge, this is the first study to genomically characterise ST80 strains and to confirm they are circulating in Ireland.

Finally, HTS was of limited usefulness in determining the genetic determinant for QD resistance in this outbreak. After comparative genomic analysis revealed this GRE outbreak was dominated by two distinct clones (ST80 and ST192), it demonstrated that there was no single, consistent genetic determinant for the QD resistance in these isolates. For the ST192 QD-resistant isolates, HTS analysis did not reveal any significant genetic differences from the QD-susceptible control isolates in the study. However, there were two genetic differences of interest present in the ST80 QD-resistant isolates which were absent in the non-ST80 QD-resistant isolates, the QD-susceptible controls and DO reference strain. These were (i) an additional 56aa leader sequence at the N-terminus of the *lsaE* gene and (ii) a potentially de-repressed transposon encoding five proteins with possible uncharacterised drug-target modification activities plus a hypothetical protein of unknown function.

Leader sequences have been implicated in inducible streptogramin resistance elsewhere. For example, streptogramins bind to the leader sequence ErmBL which stalls ribosome translation and triggers expression of *ermB* which inactivates the antibiotic through methylation [20, 21]. We found the same leader sequence of the *lsaE* gene in another *E. faecium* reference strain (TX1330) which is QD susceptible; however, this is not a truly comparable strain since it does not encode the low-level QD resistance gene *ermB* and is susceptible to glycopeptides [22]. Therefore, the 56aa leader sequence in the *lsaE* gene may possibly combine with the gene products of *ermB* or the depressed transposon of interest to generate the high-level QD resistance seen in the ST80 isolates.

From this study, we conclude that HTS was useful in demonstrating that two concurrent GRE outbreaks, both characterised by unusual QD resistance, were not directly related. It was also helpful in identifying significant genetic differences that offer plausible explanations for QD resistance in some, but not all, of the GRE in this outbreak. We conclude that there are mechanisms of QD resistance in enterococci that are not readily detectable through genomic analysis. This corresponds with the experience of others who have not been successful in using HTS to identify enterococcal mechanisms of linezolid resistance [23]. One possible explanation for this is the presence of resistance determinants that originate beyond the genome. The study of proteomics, for instance, has identified post-translational modifications that can confer antimicrobial resistance that may not be predicted by HTS [24]. As enterococcal resistance increases, QD may form part of our GRE treatment strategy in the future and further work is warranted to characterise QD resistance mechanisms at the post-genomic level.

## Appendix 1: QD resistance Genes of Interest

To identify specific QD resistance genes of interest in the assemblies, the following protein sequences were obtained from NCBI:

- gi|398085|gb|AAA26683.1|VatA VatA acetyltransferase [*Staphylococcus aureus*]
- gi|1181627|gb|AAA86871.1|VatB VatB [*Staphylococcus aureus*]
- gi|3660657|gb|AAC61671.1|VatC VatC streptogramin A acetyl transferase [Staphylococcus cohnii]
- gi|433715|gb|AAA24783.1|VatD VatD SatA streptogramin A acetyltransferase [Enterococcus faecium]
- gi|5532424|gb|AAD44719.1|VatE VatE SatG protein [Enterococcus faecium]
- gi|261607834|gb|ACX92987.1|VatG VatG streptogramin A acetyltransferase [Enterococcus faecium]
- gi|153125|gb|AAA26684.1|VgaA VgaA ATP-binding protein [Staphylococcus aureus]
- gi|2769708|gb|AAB95639.1|VgaB VgaB pristinamycin resistance protein VgaB [*Staphylococcus aureus*]
- gi|262340499|gb|ACX92986.2|VgaD VgaD ABC transporter [Enterococcus faecium]
- gi|150754|gb|AAA98259.1|VgbA VgbA virginiamycin B hydrolase [Plasmid pIP630]
- gi|3660656|gb|AAC61670.1|VgbB VgbB streptogramin B lactonase [Staphylococcus cohnii]
- gi|21492653|gb|AAA27452.2|ErmB ErmB [Enterococcus faecalis]
- gi|12659044|gb|AAK01167.1|MsrC MsrC [Enterococcus faecium]
- gi|565363052|gb|AHC08069.1|LsaE LsaE [Enterococcus faecium]
